# Cell-Based Radiotracer Binding and Uptake Inhibition Assays: A Comparison of *In Vitro* Methods to Assess the Potency of Drugs That Target Monoamine Transporters

**DOI:** 10.3389/fphar.2020.00673

**Published:** 2020-05-19

**Authors:** Marija Ilic, Julian Maier, Marion Holy, Kathrin Jaentsch, Matthias E. Liechti, Gert Lubec, Michael H. Baumann, Harald H. Sitte, Dino Luethi

**Affiliations:** ^1^ Institute of Pharmacology, Center for Physiology and Pharmacology, Medical University of Vienna, Vienna, Austria; ^2^ Department of Pharmaceutical Chemistry, Faculty of Life Sciences, University of Vienna, Vienna, Austria; ^3^ Neuroproteomics, Paracelsus Private Medical University, Salzburg, Austria; ^4^ Division of Clinical Pharmacology and Toxicology, Department of Biomedicine, University Hospital Basel and University Basel, Basel, Switzerland; ^5^ Designer Drug Research Unit, Intramural Research Program, National Institute on Drug Abuse, National Institutes of Health, Baltimore, MD, United States

**Keywords:** monoamine, transporter, radiotracer, stimulant, synaptosomes, HEK 293

## Abstract

High-affinity monoamine transporters are targets for prescribed medications and stimulant drugs of abuse. Therefore, assessing monoamine transporter activity for candidate medications and newly-emerging drugs of abuse provides essential information for industry, academia, and public health. Radiotracer binding and uptake inhibition are the gold standard assays for determining drug–transporter interaction profiles. The combined results from such assays yield a unique biochemical fingerprint for each compound. Over time, different assay methods have been developed to assess transporter activity, and the comparability of data across various assay platforms remains largely unclear. Here, we compare the effects of six well-established stimulants in two different cell-based uptake inhibition assays, one method using adherent cells and the other using suspended cells. Furthermore, we compare the data from transfected cell lines derived from different laboratories and data reported from rat synaptosomes. For transporter inhibitors, IC_50_ values obtained by the two experimental methods were comparable, but using different transfected cell lines yielded disparate results. For transporter substrates, differences between the two cell lines were less pronounced but the drugs displayed different inhibition potencies when evaluated by the two methods. Our study illustrates the inherent limitations when comparing transporter inhibition data from different laboratories and stresses the importance of including appropriate control experiments with reference compounds when investigating new drugs of interest.

## Introduction

High-affinity monoamine transporters (MATs) for serotonin (5-hydroxytryptamine [5-HT]), dopamine, and norepinephrine (SERT, DAT, and NET, respectively) are transmembrane proteins of the solute carrier 6 family that transport substrate molecules across the plasma membrane bilayer, using ion gradients (Na^+^, Cl^-^) as a driving force. These transporters contain 12 transmembrane helical domains with intracellular amino and carboxy termini (Torres et al., 2003). Although expressed mainly in the central nervous system, MATs are also present in the peripheral nervous system and other non-neuronal tissues (Rudnick, 1977; Lesch et al., 1993; Schroeter et al., 2000; Amenta et al., 2001; Ramamoorthy et al., 2011). The primary physiological role of MATs in the brain is the clearance of released monoamines from the synaptic cleft (i.e., neurotransmitter uptake), thereby terminating cell-to-cell monoamine neurotransmission (Jayanthi and Ramamoorthy, 2005).

SERT, DAT, and NET have been primary targets of medication development efforts for treating depression, anxiety, and other psychiatric disorders. For example, tricyclic antidepressants inhibit the uptake of all three monoamine transmitters, while 5-HT uptake inhibitors and 5-HT-norepinephrine uptake inhibitors are selective toward one or two transporters (Jayanthi and Ramamoorthy, 2005). In addition to approved prescription medications, most stimulant drugs of abuse act on MATs, with especially potent actions at DAT and NET. Based on their molecular mechanism of action, psychostimulants can be classified as either uptake inhibitors or releasers. Uptake inhibitors (e.g., cocaine) elevate synaptic transmitter levels by binding to the orthosteric site on transporter proteins and blocking transmitter uptake. Releasers (e.g., amphetamine) also bind to transporter proteins but are subsequently transported into neurons where they increase extracellular transmitter concentrations by disrupting intracellular vesicular storage of the transmitter or by reversing the direction of the membrane transporter flux (Cozzi et al., 1998; Scholze et al., 2000; Rothman and Baumann, 2006; Bulling et al., 2012; Eshleman et al., 2013; Rosenauer et al., 2013; Luethi et al., 2018a).

Currently, the two main assay systems used to assess uptake inhibition characteristics of new compounds are transporter-transfected human embryonic kidney (HEK) 293 cells and synaptosomes derived from rat brain. Transfected cells bear the advantage of expressing pure populations of a single human transporter of interest, whereas synaptosomes consist of sealed nerve endings which possess all of the protein machinery for transmitter synthesis, release, metabolism, and uptake. The specific experimental methods employed by different research laboratories can vary considerably, leading to high variability in transporter inhibition potency values, as reported in the scientific literature for transfected cells (Scholze et al., 2000; Verrico et al., 2007; Cameron et al., 2013; Eshleman et al., 2013; Rosenauer et al., 2013; Simmler et al., 2013; Pifl et al., 2015; Rickli et al., 2015; Mayer et al., 2016; Sandtner et al., 2016; Eshleman et al., 2017; Zwartsen et al., 2017; Luethi et al., 2018a; Luethi et al., 2018b; Luethi et al., 2019a) and for rat synaptosomes (Cozzi et al., 1998; Fleckenstein et al., 1999; Gobbi et al., 2002; Bogen et al., 2003; Escubedo et al., 2011; Hadlock et al., 2011; Lopez-Arnau et al., 2012; Baumann et al., 2013; Kolanos et al., 2015; Reith et al., 2015; Rothman et al., 2015; McLaughlin et al., 2017) (see **Table 1**). Such variability limits the comparability of findings across different laboratories, and it is often difficult to pinpoint the influence of the *in vitro* assay system, experimental protocol, or levels of protein expression, on the obtained transporter inhibition results. Therefore, the goal of the present study was to compare the transporter inhibition potential of well-studied and differently acting stimulants in two different transporter-transfected cell lines, assessed using two different established methods. Additionally, results obtained in HEK 293 cells expressing the rat transporter were compared to previously published data from rat brain synaptosomes. The transporter inhibitors cocaine and 3,4-methylenedioxypyrovalerone (MDPV), and the transporter substrates *d*-amphetamine, 3,4-methylenedioxymethamphetamine (MDMA), fenfluramine, and 4-methylmethcathinone (mephedrone) were chosen as drugs of interest. With this study, we aim to better understand how different assay variables influence the results obtained, and examine whether the identification of certain trends in the data might facilitate a better comparison of results.

**Table 1:** Range of previously reported IC_50_ values for neurotransmitter uptake inhibition.

	Cocaine	MDPV	*d*-Amphetamine	MDMA	Fenfluramine	Mephedrone
IC_50_ (µM)						
	**SERT**					
HEK 293	0.3–2.4^1^	1.4–12^2^	> 10–110^3^	0.1–121^4^	1.1^5^	0.5–26^6^
Synaptosomes	0.3–1.0^7^	3.3^8^	3.4–8.0^9^	0.1–4.1^10^	0.5–5^11^	0.3–0.6^12^
	**DAT**					
HEK 293	0.3–1.3^13^	0.01–0.05^14^	1.3–7.5^15^	0.2–43^16^		0.1–98.8^17^
Synaptosomes	0.2–1.0^18^	0.004–0.005^19^	0.093–0.094^20^	1.0–3.9^21^	13.8–21.5^22^	0.5–1.1^23^
	**NET**					
HEK 293	0.2–1.9^24^	0.02–0.04^25^	0.07–1.5^26^	0.02–12.4^27^		0.05–6.8^28^
Synaptosomes	0.3–0.4^29^	0.02–0.03^30^	0.07^31^	0.5^32^	8.0^33^	0.2–0.5^34^

Each concentration range was derived from various studies (indicated by superscripts). Baumann et al. (2013): ^7,8,9,10,12,18,19,20,21,23,29,30,31,32,34^, Hadlock et al. (2011): ^7,18,10,21,12,23^, Reith et al. (2015): ^19^, Bogen et al. (2003): ^10,21^, Kolanos et al. (2015): ^18,19,29,30^, Rickli et al. (2015): ^2,3,4,14,15,16,25,26,27^, Cameron et al. (2013): ^13,14,17^, Lopez-Arnau et al. (2012): ^12,23,34^, Rosenauer et al. (2013): ^3,4,6,15,16,17,26,27,28^, Cozzi et al. (1998): ^11,22,33^, Luethi et al. (2018a): ^1,13,24^, Rothman et al. (2015): ^7,18,29^, Escubedo et al. (2011): ^10,21^, Luethi et al. (2018b): ^6,17,28^, Sandtner et al. (2016): ^4^, Eshleman et al. (2013): ^1,2,4,6,13,14,16,17,24,25,27,28^, Luethi et al. (2019a): ^2,4,14,16,25,27^, Scholze et al. (2000): ^5^, Eshleman et al. (2017): ^1,4,13,16,24,27^, Mayer et al. (2016): ^6,17,28^, Simmler et al. (2013): ^1,2,3,4,6,13,14,15,16,17,24,25,26,27,28^, Fleckenstein et al. (1999): ^7,9,11,18,20,22^, McLaughlin et al. (2017): ^12,23,34^, Verrico et al. (2007): ^4,16,27^, Gobbi et al. (2002): ^10^, Pifl et al. (2015): ^4,6,16,17,27,28^, Zwartsen et al. (2017): ^1,3,4,13,15,16,24,26,27^, MDPV, 3,4-methylenedioxypyrovalerone; MDMA, 3,4-methylenedioxymethamphetamine; HEK, human embryonic kidney.

## Materials and Methods

Transporter inhibition was assessed in two different laboratories, one performing experiments on adherent cells (referred to as “method 1”) and the other performing experiments on cells in suspension (referred to as “method 2”). Each of the laboratories independently established stable cell lines expressing human isoforms of SERT, DAT or NET using their stock of HEK 293 cells. For reasons of simplicity, human MAT expressing cells established in Vienna and Basel, will henceforth be referred to as “cell line 1” and “cell line 2,” respectively. Both cell lines have been previously used to assess the pharmacological profiles of stimulants in multiple studies (**Table 1**). The transporter-transfected cell lines were furthermore exchanged between laboratories in order to compare differences between methods as well as differences between transfected cell lines. In addition, stable rat transporter-transfected HEK 293 cells (referred to as “cell line 3”) were prepared for comparison of data derived from rat transporter-transfected cells with previously published data from rat brain synaptosomes.

### Drugs

Due to the limited availability of certain drugs in different countries, the contributing laboratories obtained some of the test drugs from different sources. The following test drugs were included: cocaine hydrochloride (Sigma Aldrich, Vienna, Austria and Lipomed, Arlesheim, Switzerland), MDPV hydrochloride (Lipomed), *d*-amphetamine sulfate (Sigma Aldrich) and *d*-amphetamine hydrochloride (Lipomed), fenfluramine hydrochloride (Lipomed), MDMA hydrochloride (Lipomed), and mephedrone hydrochloride (Lipomed).

### Chemicals and Reagents

Buffers used for the uptake inhibition, uptake saturation, and radioligand binding experiments were as follows: Krebs HEPES buffer (KHB; 10 mM HEPES, 120 mM NaCl, 3 mM KCl, 2 mM CaCl_2_ ∙ 2H_2_O, 2 mM MgCl_2_ ∙ 6H_2_O, pH 7.3) and Krebs-Ringer Bicarbonate buffer (KRB; Sigma-Aldrich, Buchs, Switzerland). KHB used for binding experiments at DAT additionally contained 10 µM ZnCl_2_. The lysis buffer used in method 2 was composed of 0.05 M Tris–HCl, 50 mM NaCl, 5 mM EDTA, and 1% NP-40 in purified water. Radiotracers used in method 1 and uptake saturation experiments were purchased from American Radiolabeled Chemicals, Saint Louis, MO, USA ([^3^H]-1-methyl-4-phenylpyridinium ([^3^H]-MPP^+^), 80 Ci/mmol) or from Perkin Elmer, Boston, MA, USA ([^3^H]-dopamine, 19.1 Ci/mmol and [^3^H]-5-HT, 36.5 Ci/mmol). Radiotracers used in method 2 were purchased from Perkin Elmer, (Schwerzenbach, Switzerland; [^3^H]-norepinephrine, 10.0 Ci/mmol and [^3^H]-dopamine, 32.6 Ci/mmol) or Anawa, Zürich, Switzerland ([^3^H]-5-HT, 80.0 Ci/mmol). [^3^H]-CFT (WIN 35,428), 82.9 Ci/mmol, used in binding experiments was obtained from Perkin Elmer Boston, MA, USA.

### Cell Culture

HEK 293 cells used to create cell lines stably expressing MAT proteins in Vienna, Austria were purchased from LGC Standards GmbH, Wesel, Germany. Human isoforms of DAT and SERT were transfected using jetPRIME transfection reagent (VWR International GmbH, Vienna, Austria) according to manufacturer's instructions. The human isoform of NET and rat isoforms of DAT, NET, and SERT were transfected using the CaPO_4_ method described in detail elsewhere (Mayer et al., 2017). Cells were cultured in Dulbecco's modified Eagle's medium (DMEM; Sigma-Aldrich) with high glucose (4.5 g/l) and L-glutamine (584 mg/l) supplemented with 10% fetal calf serum (FCS; Biowest) and penicillin/streptomycin mixture (50 mg/l) (Sigma-Aldrich) at 37 °C in a 5% CO_2_ humidified atmosphere. HEK 293 cells, stably transfected with NET, DAT or SERT, were selected using Geneticin (G418; Merck, Darmstadt, Germany) and subsequently grown in 10-cm tissue culture dishes. Upon reaching approximately 80% confluence, the cells were washed once with 6 ml of phosphate-buffered saline (PBS; 2.7 mM KCl, 137 mM NaCl, 1.5 mM KH_2_PO_4_, 4.3 mM Na_2_HPO_4_ ∙ 2H_2_O, pH 7.4) and detached with 1 ml of trypsin-EDTA (Sigma-Aldrich). The cell trypsinization was stopped after 3 min by adding 9 ml of pre-warmed DMEM. The cells were seeded onto poly-D-lysine-coated (PDL; 0.05 mg/ml; Sigma-Aldrich) 96-well culture plates at the density of 0.2 × 10^6^ cells/ml 24 h prior to the experiment and grown as monolayers.

HEK 293 cells (Invitrogen, Zug, Switzerland) stably transfected with the human NET, DAT, or SERT using the CaPO_4_ method as previously described (Tatsumi et al., 1997) were created in Basel, Switzerland. Cells were cultured in DMEM (Gibco, Life Technologies, Zug, Switzerland) supplemented with 10% fetal bovine serum (Gibco) and 250 μg/ml Geneticin (Gibco) in T75 (SERT) or T150 (NET and DAT) tissue culture flasks (Techno Plastic Products, Trasadingen Switzerland).

### Method 1: Uptake Inhibition in Adherent Transporter-Transfected HEK Cells

Assays were performed as previously described by Mayer et al. (2017). On the day of the experiment, cells were washed once with 100 µl of room temperature KHB and then incubated for 5 min at room temperature in 50 µl of the same buffer containing various concentrations of the test drugs, vehicle control, or monoamine-specific inhibitors (at concentrations of at least 100-fold their K_i_). To initiate uptake, the preincubation buffer was exchanged for a buffer containing various concentrations of the test drugs, vehicle control, or monoamine-specific inhibitors with the addition of 20 nM [^3^H]-MPP^+^, 200 nM [^3^H]-dopamine, or 100 nM [^3^H]-5-HT. The uptake was carried out at room temperature for 1 min (for DAT and SERT) or 3 min (for NET) and the reaction was subsequently stopped by rapid removal of the uptake buffer and washout with ice-cold KHB. Thereafter, 300 µl of 1% SDS was added to the wells and cell lysates were transferred into 2 ml of scintillation cocktail. The amount of accumulated [^3^H]-substrate was determined by liquid scintillation counting on a Packard Tri-Carb 2300 TR liquid scintillation analyzer.

### Method 2: Uptake Inhibition in Resuspended Transporter-Transfected HEK Cells

Upon 70%–90% confluence, HEK 293 cells cell lines stably expressing NET, DAT, or SERT were washed once with 10 ml of PBS (Gibco) and detached with 2 ml (SERT) or 4 ml (NET and DAT) of 0.05% Trypsin-EDTA (Gibco). After 2 min, the trypsinization was stopped with 10 ml (SERT) or 8 ml (NET and DAT) of pre-warmed culture medium, the cell suspension was transferred into a 15-ml polypropylene tube (Techno Plastic Products), and the cells were then centrifuged at 1100 revolutions per minute (rpm) for 2 min at ambient temperature with a Heraeus Multifuge X1R (Thermo Scientific, Reinach, Switzerland). Thereafter, the medium was removed, and the cells were washed once with KRB, centrifuged again, and then resuspended in KRB at a density of 3 × 10^6^ cells/ml. For [^3^H]-dopamine uptake experiments, the buffer was additionally supplemented with 0.2 mg/ml ascorbic acid. The cell suspension (100 μl per well) was transferred into a round bottom 96-well polypropylene storage plate (Corning, Amsterdam, the Netherlands) and incubated in triplicate with 25 μl of KRB containing the test drugs in the range of 1 nM to 900 μM final assay concentration (11–13 point 1:3 serial dilution), vehicle control, or monoamine-specific inhibitors (10 μM nisoxetine for NET, 10 μM mazindol for DAT, and 10 μM fluoxetine for SERT) for 10 min at 450 rpm and ambient temperature on a rotary shaker. To initiate uptake transport, 50 μl of [^3^H]-norepinephrine, [^3^H]-dopamine or [^3^H]-5-HT dissolved in KRB were added at a final concentration of 5 nM for additional 10 min. Thereafter, 100 μl of the cell suspension was transferred into 0.5-ml microtubes (Sarstedt, Nümbrecht, Germany) that contained 50 μl of 3 M KOH covered with 200 μl silicon oil [1:1 (w/w) mixture of silicon oil types AR 20 and AR 200; Sigma-Aldrich]. The tubes were centrifuged with a Mikro 220 centrifuge (Hettich, Bäch, Switzerland) for 3 min at 16,550 × g to transport the cells through the silicone oil phase into the KOH. The tubes were frozen in liquid nitrogen and the cell pellets were cut from the bottom of the tubes and transferred into 6 ml scintillation vials (Perkin-Elmer) that contained 0.5 ml lysis buffer. The samples were shaken for 1 h at 700 rpm before 3 ml scintillation fluid (Ultima Gold; Perkin Elmer) was added. After 1-h equilibration time, monoamine uptake was quantified by liquid scintillation counting on a Packard Tri-Carb 1900 TR liquid scintillation analyzer. Nonspecific uptake in the presence of selective inhibitors was subtracted from the total counts.

### Radioligand Uptake and Binding in Adherent Transporter-Transfected HEK Cells

The cells were prepared as described for adherent cells in the *Cell Culture* section, washed once with 100 µl of room temperature KHB and incubated for 1 min (for DAT and SERT) and 3 min (for NET) at room temperature in 50 µl of the KHB containing various concentrations of the [^3^H]-substrate. The dilution row of [^3^H]-substrate was created by mixing various concentrations of non-tritiated substrates with a constant amount of [^3^H]-substrates (20 nM [^3^H]-MPP^+^, 200 nM [^3^H]-dopamine, or 200 nM [^3^H]-5-HT). 100% of uptake was obtained in the presence of [^3^H]-substrate only and non-specific uptake was determined in the presence of monoamine-specific inhibitors.

For the measurement of inhibitor binding, cells were washed once with 100 µl of room temperature KHB and incubated for 30 min at room temperature in 50 µl of KHB containing various concentrations of inhibitors and 10 nM [^3^H]-CFT. Saturation binding was performed in KHB containing various concentrations of non-tritiated β-CFT and 10 nM [^3^H]-CFT. After the incubation, cells were washed twice with 100 µl of ice-cold KHB and lysed with 1% SDS. The amount of released tritiated substrate was quantified by liquid scintillation counting on a Packard Tri-Carb 2300 TR liquid scintillation analyzer.

### Calculations and Statistics

Nonlinear regression analysis (GraphPad Prism 5 software, CA, USA) was used for the calculation of IC_50_ values. To determine kinetic parameters (i.e., K_m_, V_max_, K_d_, and B_max_) the Michaelis–Menten equation was fitted to the data using the nonlinear least-squares regression analysis. Statistical significance was analyzed using Student's *t*-test or one-way ANOVA, as appropriate. Differences were considered to be significant when P < 0.05. All statistical data are included in **Supplementary Tables 1–7**.

## Results

**Figure 1:**
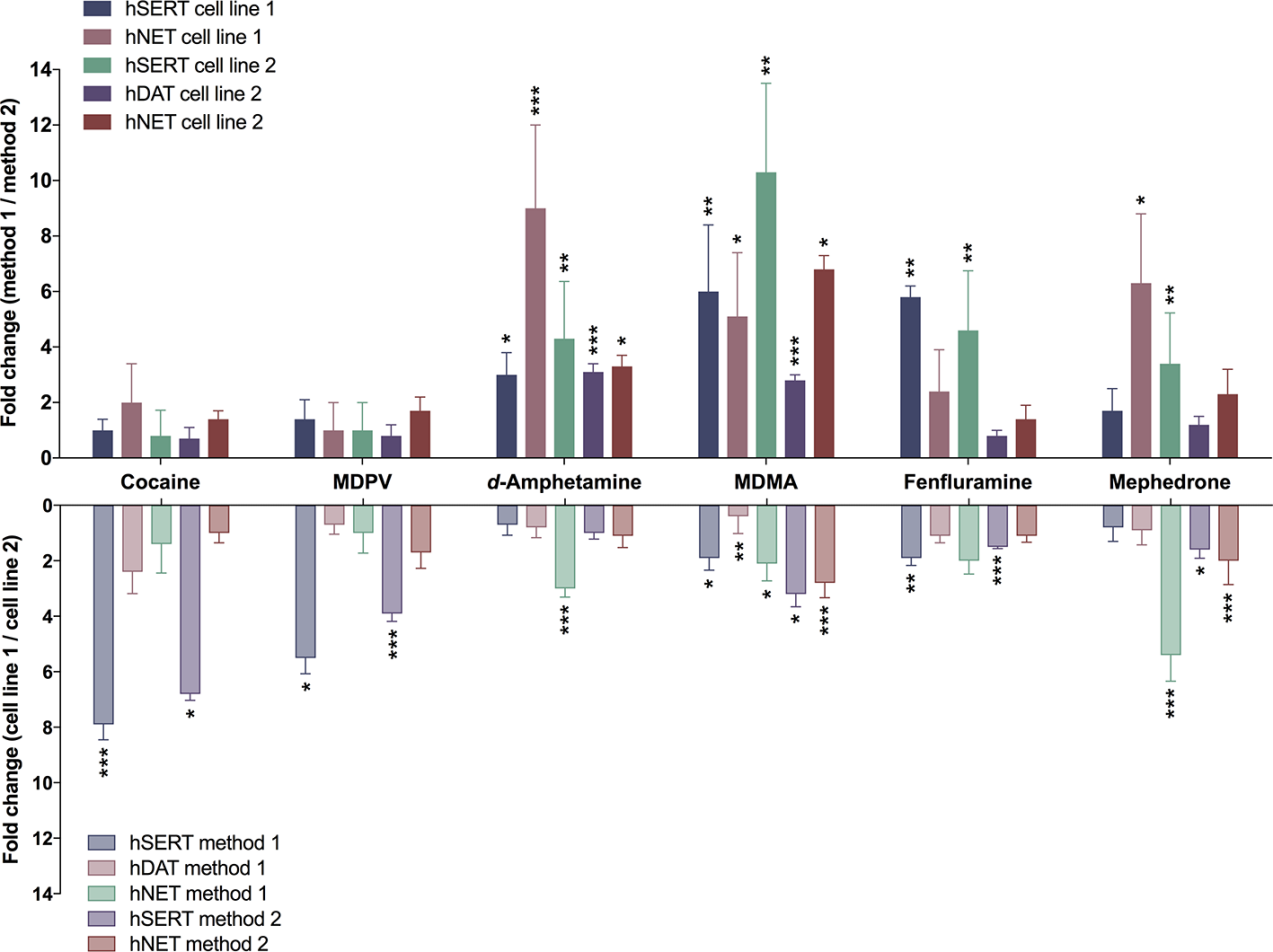
Differences in transporter inhibition when assessed with different methods or in different cell lines. Bars represent fold-change of IC_50_ values determined either with different methods (upper section) or with different cell lines [lower section; method 1 (adherent cells); method 2 (cells in suspension)]. Ratios were calculated from the IC_50_ values present in **Table 2**. Error bars represent error propagation from the division of two values. Asterisks indicate significant differences between two methods or two (cell lines used (*P < 0.05, **P < 0.01, ***P < 0.001; unpaired two-tailed t-test; **Supplementary Tables 1** and **2**).

**Table 2** shows IC_50_ values for cocaine, MDPV, *d*-amphetamine, MDMA, fenfluramine, and mephedrone assessed with two different methods in two different HEK cell lines, stably expressing human MATs. Corresponding sigmoidal uptake inhibition curves are provided in **Supplementary Figure 1**. **Figure 1** illustrates the fold-change when transporter inhibition was assessed with different methods or different cell lines. For one of the DAT-transfected cell lines, the assay procedure using resuspended cells manifested in non-sigmoidal uptake curves; IC_50_ values could therefore not be assessed and are lacking in the evaluation of the study.

**Table 2:** Comparison of two uptake inhibition methods in different HEK 293 cell lines stably expressing human MATs.

	Cocaine	MDPV	*d*-Amphetamine	MDMA	Fenfluramine	Mephedrone
IC_50_ ± SD (µM)						
**Cell line 1**	**SERT**					
Method 1	8.7 ± 2.7	55.4 ± 27.1	151.4 ± 39.4	32.6 ± 9.0	13.9 ± 1.0	11.7 ± 5.1
Method 2	8.9 ± 1.9	39.0 ± 3.6	50.0 ± 4.4	5.4 ± 1.6	2.4 ± 0.0	6.8 ± 0.8
	**DAT**					
Method 1	1.2 ± 0.8	0.02 ± 0.001	3.6 ± 1.3	19.6 ± 12.0	69.7 ± 15.6	6.1 ± 2.8
Method 2	NA	NA	NA	NA	NA	NA
	**NET**					
Method 1	1.0 ± 1.0	0.05 ± 0.03	0.9 ± 0.06	5.6 ± 1.9	15.6 ± 6.8	3.8 ± 1.5
Method 2	0.5 ± 0.1	0.05 ± 0.02	0.1 ± 0.03	1.1 ± 0.1	6.6 ± 0.9	0.6 ± 0.03
**Cell line 2**	**SERT**					
Method 1	1.1 ± 0.5	10.0 ± 3.0	216.8 ± 58.8	17.5 ± 6.1	7.4 ± 1.9	14.1 ± 3.4
Method 2	1.3 ± 0.1	10.0 ± 2.6	51.0 ± 10.2	1.7 ± 0.6	1.6 ± 0.1	4.2 ± 1.2
	**DAT**					
Method 1	0.5 ± 0.2	0.03 ± 0.01	4.7 ± 0.3	50.1 ± 3.0	62.8 ± 7.4	6.6 ± 1.7
Method 2	0.7 ± 0.1	0.04 ± 0.01	1.5 ± 0.4	18.0 ± 3.6	81.0 ± 17.1	5.7 ± 1.3
	**NET**					
Method 1	0.7 ± 0.2	0.05 ± 0.02	0.3 ± 0.1	2.7 ± 1.4	7.9 ± 1.5	0.7 ± 0.6
Method 2	0.5 ± 0.1	0.03 ± 0.01	0.09 ± 0.02	0.4 ± 0.04	5.8 ± 2.4	0.3 ± 0.03

[^3^H]-substrate uptake inhibition assays were conducted as described in the Materials and Methods section. IC_50_ values represent means ± SD of at least three independent experiments; MAT, monoamine transporter; NA, not assessed.

### Comparison of Different Assay Procedures Using Transporter-Transfected HEK Cells

The different assay procedures resulted in no statistically significant differences in IC_50_ values for the transporter inhibitors cocaine and MDPV when measured in the same transfected cell line (**Table 2**, **Supplementary Table 1**, and **Supplementary Figure 1**). The IC_50_ values for the transporter substrates *d*-amphetamine and MDMA were significantly different for all transporters in both cell lines, with 3- to 10-fold lower values for the method using resuspended cells. Fenfluramine displayed higher potency at all transporters when assessed with the method using resuspended cells, which was statistically significant only in the case of both SERT cell lines. IC_50_ values for mephedrone were either unaltered or lower (cell line 1—NET and cell line 2—SERT) when cells in suspension were used in the assay.

### Comparison of Different Transporter-Transfected Cells Lines

For cocaine, MDPV, and fenfluramine, IC_50_ values were significantly different only in the case of SERT cell lines (**Table 2**, **Supplementary Table 2**, and **Supplementary Figure 1**). MDMA values were significantly different across all of the examined transporters and *d*-amphetamine values were significantly different only in the case of NET when assayed with method 1. The IC_50_ values for mephedrone differed only at NET when assessed with both methods and at SERT when assessed with method 2.

The IC_50_ values of two sets of adherent cell lines expressing human transporters and adherent cell lines expressing rat transporters (cell line 3) were comparable (**Table 5**, **Supplementary Table 3**, and **Supplementary Figure 2**) only for cocaine and MDPV when DAT was assayed and for mephedrone when SERT was assayed.

Inhibition of [^3^H]-CFT binding at NET, DAT, and SERT (human and rat species) is shown in **Table 3**. The most striking difference in IC_50_ values was observed for the inhibition of [^3^H]-CFT binding by cocaine at SERT (8-fold) followed by inhibition by *d*-amphetamine at NET and MDPV at SERT (3-fold difference). Approximately half of the IC_50_ values did not differ among two cell lines and the rest differed by a factor of 2 or less (**Supplementary Table 4**, and **Supplementary Figure 4**). None of the human DAT IC_50_ values differed, however, when compared to cells expressing rat DAT, a significant difference was observed only for mephedrone (**Supplementary Table 5**). The obtained values for human SERT cell lines did not differ significantly when fenfluramine and mephedrone were assayed but differed significantly for all substances when all three cell lines were compared (**Supplementary Figure 5**). The IC_50_ values for NET were comparable only for inhibitors, namely cocaine and MDPV, among all 3 cell lines.

**Table 3:** Binding inhibition of [^3^H]-CFT to adherent HEK 293 cells expressing human and rat MATs.

	Cocaine	MDPV	*d*-Amphetamine	MDMA	Fenfluramine	Mephedrone
IC_50_ ± SD (µM)						
**Cell line 1**						
hSERT	0.25 ± 0.03	1.4 ± 0.3	57.5 ± 11.2	4.9 ± 0.9	1.8 ± 0.8	5.0 ± 1.0
hDAT	0.24 ± 0.09	0.014 ± 0.002	5.2 ± 3.6	34.8 ± 6.2	24.7 ± 5.9	3.4 ± 0.4
hNET	0.16 ± 0.04	0.027 ± 0.005	0.6 ± 0.1	4.4 ± 0.8	4.4 ± 0.3	0.8 ± 0.1
**Cell line 2**						
hSERT	1.9 ± 0.4	3.6 ± 0.6	25.6 ± 5.3	8.1 ± 1.6	1.15 ± 0.02	4.9 ± 1.2
hDAT	0.23 ± 0.03	0.03 ± 0.01	9.9 ± 2.6	33.0 ± 9.6	24.2 ± 2.4	3.4 ± 0.6
hNET	0.18 ± 0.02	0.03 ± 0.01	0.2 ± 0.1	1.8 ± 0.7	3.2 ± 1.8	0.4 ± 0.1
**Cell line 3**						
rSERT	0.17 ± 0.03	1.3 ± 0.6	22.5 ± 5.8	2.8 ± 0.4	2.8 ± 0.1	2.4 ± 0.7
rDAT	0.31 ± 0.09	0.02 ± 0.01	7.0 ± 2.9	28.4 ± 8.9	29.6 ± 7.7	5.6 ± 1.1
rNET	0.3 ± 0.2	0.03 ± 0.01	0.04 ± 0.02	0.4 ± 0.2	1.5 ± 0.5	0.2 ± 0.1

[3H]-CFT binding assays were conducted as described in Radioligand Uptake and Binding in Adherent Transporter-Transfected HEK Cells. IC_50_ values represent means ± SD of at least three independent experiments.

### Saturation Kinetics Comparison of Different Transporter-Transfected Cell Lines

The K_m_ and V_max_ values acquired by the same method did not differ significantly at the two different human DAT-transfected cell lines (**Table 4**, **Supplementary Table 6**, and **Supplementary Figure 3**). However, although K_m_ at different human SERT-transfected cell lines did not differ significantly, values for V_max_ were 3-fold different. A similar trend was observed in the case of the two human NET-expressing cell lines, for which K_m_ and V_max_ values significantly differed 3- and 2-fold, respectively. K_m_ and V_max_ values of rat SERT were comparable to the K_m_ and V_max_ values of human SERT expressing cell line 2 and significantly (2- to 3-fold) lower than K_m_ and V_max_ values of human SERT expressing cell line 1 (**Supplementary Table 7**). K_m_ and V_max_ values of rat NET were distinctively (up to 15-fold) lower when compared to the two human NET-expressing cell lines. Values for rat DAT were 2-fold higher than in both cell lines expressing human DAT. The binding site density (B_max_) for β-CFT binding was similar for DAT and NET among all three cell lines (**Supplementary Figure 3**). The determined B_max_ for human SERT assessed in cell line 1 was 30-fold higher than the B_max_ values of the other two SERT cell lines. The K_d_ values obtained for all three DAT and NET cell lines did not differ significantly, in contrast to SERT cells, for which the K_d_ values differed 10-fold.

**Table 4:** Kinetic parameters for the uptake and binding of radioligands in adherent transporter-transfected HEK 293 cells.

	K_m_ ± SD (µM)	V_max_ ± SD (pmol/min/10^6^ cells)	K_d_ ± SD (µM)	B_max_ ± SD (pmol/min/10^6^ cells)
**Cell line 1**				
hSERT	8.1 ± 3.0	483 ± 57	0.12 ± 0.03	112 ± 31
hDAT	4.7 ± 3.3	323 ± 98	0.1 ± 0.08	0.5 ± 0.1
hNET	4.6 ± 1.2	205 ± 17	0.05 ± 0.02	0.4 ± 0.1
**Cell line 2**				
hSERT	4.1 ± 1.0	160 ± 16	1.2 ± 0.4	3.6 ± 1.9
hDAT	5.5 ± 0.8	370 ± 68	0.051 ± 0.007	0.7 ± 0.2
hNET	1.4 ± 0.7	104 ± 9	0.07 ± 0.02	0.4 ± 0.1
**Cell line 3**				
rSERT	3.4 ± 1.5	179 ± 59	1.13 ± 0.07	3.0 ± 0.9
rDAT	8.8 ± 3.9	604 ± 23	0.07 ± 0.02	1.0 ± 0.2
rNET	0.3 ± 0.1	18.1 ± 4.7	0.13 ± 0.07	0.4 ± 0.1

Radioligand uptake and binding assays were conducted as described in Radioligand Uptake and Binding in Adherent Transporter-Transfected HEK Cells. Values represent means ± SD of at least three independent experiments.

### Comparison of Rat Transporter-Transfected HEK Cells and Rat Synaptosomes

The reported IC_50_ values determined in rat synaptosomes were typically lower compared to results in rat transporter-transfected HEK cells except for mephedrone for which the potency was higher at NET when assayed in cells (**Table 5**). The most pronounced differences in potencies were observed for SERT expressing systems, for which the IC_50_ values ranged from 10- to 106-fold. The differences for DAT expressing systems ranged from 7- to 26-fold, while NET expressing systems showed the smallest variability (2- to 8-fold difference). Notably, the IC_50_ values for the binding inhibition by cocaine at all rat transporter-transfected cell lines showed typically less than a 2-fold difference when compared to the IC_50_ values of the uptake inhibition in rat synaptosomes and 2.5- and 4-fold difference for SERT and NET cell lines, respectively, when MDPV was used (**Tables 3** and **5**). This indicates a higher level of comparability for pure inhibitors.

**Table 5:** Radioligand uptake inhibition in rat synaptosomes and HEK 293 cell lines stably expressing rat MATs.

	Cocaine	MDPV	*d*-Amphetamine	MDMA	Fenfluramine	Mephedrone
IC_50_ ± SD (µM)						
	**SERT**					
Synaptosome	0.31 ± 0.17^1^	3.3 ± 0.3^1^	3.4 ± 0.3^1^	0.24 ± 0.01^2^	0.27 ± 0.07^2^	0.42 ± 0.03^1^
Cell	4.3 ± 2.1	31.6 ± 7.6	103.5 ± 21.5	13.8 ± 4.7	12.7 ± 2.8	13.1 ± 3.3
	**DAT**					
Synaptosome	0.21 ± 0.02^1^	0.004 ± 0.001^1^	0.09 ± 0.02^1^	1.6 ± 0.6^2^	23.7 ± 1.3^2^	0.8 ± 0.1^1^
Cell	2.2 ± 1.6	0.04 ± 0.03	2.3 ± 1.3	24.8 ± 10.8	140.5 ± 14.2	12.0 ± 3.4
	**NET**					
Synaptosome	0.29 ± 0.03^1^	0.026 ± 0.008^1^	0.07 ± 0.02^1^	0.5 ± 0.2^2^	1.9 ± 0.2^2^	0.49 ± 0.07^1^
Cell	2.4 ± 2.0	0.07 ± 0.02	0.13 ± 0.03	0.9 ± 0.3	6.0 ± 2.2	0.3 ± 0.2

1 Value reported previously (Baumann et al., 2013)

2 Value reported previously (Rothman et al., 2003)

3 [^3^H]-substrate uptake inhibition assays were conducted as described in Method 1: Uptake Inhibition in Adherent Transporter-Transfected HEK Cells. IC_50_ values represent means ± SD of at least three independent experiments.

## Discussion

In the present study, we investigated the effects of different assay conditions and different transfected cell lines on kinetic constants and functional activities of six well-established psychostimulants. Each of the two laboratories involved used its own assay design and applied it to their cell lines, and cell lines provided by the collaborative laboratory, containing the identical human MATs. In addition, we compared the inhibitory potency of these substances among cell lines expressing rat MATs and to previously published data from rat synaptosomes.

The transporter inhibition profiles of cocaine and MDPV reported here agree with previous work and suggest little difference in IC_50_ values for pure inhibitors at all three transporters assessed with different cell-based methods (**Figure 1** and **Table 2**). However, the results for substrate-type releasers differ remarkably depending on the specific assay set-up, with up to 10-fold differences in the IC_50_ values observed for some of the substances. It should be stressed that, although uptake incubation times differed among the two assays, the chosen uptake times are within the linear range of transport (up to 10 min for all three transporters) and should not affect the outcome (Banerjee et al., 1987; Lee et al., 1996; Sitte et al., 2001). One possible source of observed variability might be the different final concentration of tritiated substrate. In method 2, substantially lower concentrations of tritiated substrate were used and the consequence of that might be substrate depletion induced by some of the substrate-type substances tested. Consistently lower IC_50_ values observed in method 2 reinforce this interpretation. These findings strongly undermine the comparability of results obtained with different methods. The different inhibition potencies observed with various methods are illustrated by the example of mephedrone; while some *in vitro* pharmacological studies report that mephedrone has greater (3- to 10-fold) dopaminergic activity over serotonergic activity (Eshleman et al., 2013; Pifl et al., 2015; Mayer et al., 2016), others found it to display similar inhibition potency at both transporters (Hadlock et al., 2011; Simmler et al., 2013; Luethi et al., 2018b). The issue of MAT selectivity is a key factor when characterizing stimulant drugs. NET and DAT inhibition potencies correlate with clinical potencies of stimulants (Luethi and Liechti, 2018), and the DAT/SERT inhibition ratio is a predictor of the reinforcing effects and abuse liability of a substance (Baumann et al., 2000).

The *in vitro* findings presented here are variable regarding whether or not mephedrone exerts more dopaminergic *vs.* serotonergic activity, but findings from controlled mephedrone administration in humans demonstrate that mephedrone has cardiovascular and neurological effects mimicking the effects of MDMA, an established substrate at SERT (Papaseit et al., 2016). In a recent study, Olesti et al. showed that oral administration of mephedrone to human subjects can significantly elevate plasma concentrations of 5-HT (Olesti et al., 2019). MAT substrates are able to release 5-HT from platelets *via* a SERT-mediated mechanism, which is a proxy for SERT-mediated 5-HT release in the brain (Yubero-Lahoz et al., 2013). Elevations of plasma 5-HT induced by mephedrone in humans are highly correlated with circulating plasma concentrations of the drug. Thus, available human data show that mephedrone, like MDMA, displays substantial serotonergic activity which suggests non-selective substrate activity at MATs.

Compared to the use of two different methods, the differences in IC_50_ values showed no apparent pattern when compounds were profiled with the same method in two different stably transfected cell lines (**Figure 1** and **Table 2**). Interestingly, whereas the IC_50_ values for the transporter blockers cocaine and MDPV were almost identical when using two different methodological approaches, the difference in SERT inhibition was more pronounced for these two substances when different cell lines were used. The distinct IC_50_ values for transporter blockers at SERT might be due to individual saturation kinetics. Namely, uniform K_m_ values and the 3-fold change in V_max_ values coincide with greater variability in IC_50_ values at SERT when using the same method (**Table 4**). Surprisingly, not all substrate-type substances were affected by this parameter. The cell line choice did not affect *d*-amphetamine potency at SERT when assayed with either method, moreover, among all the substrates the affinity of *d*-amphetamine for SERT was the lowest. A similar trend in NET saturation kinetics parameters was observed for NET cell lines. However, the highest variability in the IC_50_ values was observed for releaser-type compounds. In contrast, there was no variability in the potencies of inhibitors cocaine and MDPV. DAT cell lines did not show any variability in K_m_ and V_max_ values but contrary to SERT, the fold change difference in inhibition potencies was observed only in the case of some of the releasers. It is difficult to explain the variability of IC_50_ values based solely on kinetic parameters because there was more similarity observed for DAT and NET inhibition profiles, and kinetic parameters varied only for SERT and NET. The similar inhibition profiles of DAT and NET might be explained by the higher amino acid sequence homology identity within the substrate-binding site (86%) of these catecholamine transporters, whereas the sequence homology with SERT binding site is much lower (57% and 68% compared to DAT and NET, respectively) (Koldso et al., 2013).

The comparison of inhibition potencies of substances at cell lines expressing human or rat MATs (**Tables 2**, **5**) resulted in considerable variability when the adherent-cell method was used. These findings suggest that a straightforward comparison of human and rat isoforms of MATs can not be made.

The IC_50_ values for the inhibition of [^3^H]-CFT binding were more consistent compared to the IC_50_ values for the uptake inhibition. Similar to uptake inhibition, the most notable difference in the IC_50_ values for binding inhibition was observed for SERT cell lines (**Table 3**). This observation coincides with striking differences in K_d_ and B_max_ values for β-CFT binding at SERT where the K_d_ was 10-fold higher for cell line 2 and the B_max_ was 31-fold higher for cell line 1. However, the inhibition of [^3^H]-CFT binding at SERT varied among different cell lines in the case of both inhibitors and two releasers (*d*-amphetamine and MDMA). The next highest variation between two cell lines was observed in the case of norepinephrine uptake inhibition induced by *d*-amphetamine, MDMA, and mephedrone, even though there was no difference in K_d_ and B_max_ values for β-CFT binding. There was no difference in β-CFT binding inhibition among all substances when two different DAT cell lines were assayed. This is in agreement with no differences in K_d_ and B_max_ values for the two DAT cell lines.

When the two human MATs expressing cell lines were compared to rat MATs expressing cell lines, transporter density varied for SERT and DAT (**Table 4**). However, the pattern of inhibition potency variabilities was similar to the variability between human cell lines. Moreover, even though the transporter density did not differ between human and rat NET cell lines, the difference in β-CFT binding inhibition was observed for all substrate-type compounds. This assay, together with the uptake inhibition assay, suggests that the variation in transporter expression levels contributes to the variability in previously published data.

A puzzling finding of this study was the fact that when using the method with cells in suspension, only one of the two stably DAT-transfected cell lines could be used, whereas with the other cell line no sigmoidal uptake curve was observed for any of the substances. The same phenomenon has recently been observed for catechol metabolites of MDMA and 3,4-methylenedioxymethcathinone (methylone) (Luethi et al., 2019a). A possible explanation for this phenomenon is the structural similarity between the catechol metabolites in question and dopamine (Luethi et al., 2019a). In the same study, sigmoidal uptake curves were observed for the catechol metabolite of the DAT blocker MDPV, indicating that the observed phenomenon is limited to DAT substrates (Luethi et al., 2019a). However, in this study, no dopamine uptake inhibition could be assessed for any of the six compounds, independent of their activity as substrates or structural similarity to dopamine.

A literature review of studies performed in rat brain synaptosomes revealed some variability in MAT uptake inhibition potencies as observed in studies using transfected HEK cells (**Table 5**). The most striking observation is that all investigated stimulants inhibited MATs in rat brain synaptosomes at much lower concentrations when compared to rat transporters overexpressed in HEK cells (i.e., all drugs appeared more potent in synaptosomes). This discrepancy was most pronounced in the case of SERT. For instance, MDMA inhibited synaptosomal rat SERT with 110-fold higher potency when compared to its recombinant counterpart expressed in non-neuronal cells. The rest of the stimulants showed at least 10-fold differences in potency at rat SERT expressed *in situ* versus rat SERT expressed in HEK cells. Similarly, all stimulants exhibited higher potencies at synaptosomal rat DAT and rat NET compared to the respective recombinant transporters expressed in HEK cells. An exception to this was mephedrone, which exhibited higher potency at recombinant rat NET compared to synaptosomal rat NET. The observed discrepancies might be related to fundamental differences between synaptosomes and intact cells. Synaptosomes are tissue homogenates containing an enriched preparation of sealed nerve endings, which may facilitate a higher probability for drug–transporter interactions. Furthermore, transporter proteins expressed in synaptosomes are surrounded by neuron-specific protein partners that are not present in HEK cells. Finally, procedural differences between the synaptosome experiments *vs.* cell-based experiments could be a factor. For example, the incubation times for MAT uptake assays in adherent cells are 1–3 min whereas incubation times for synaptosomes are 5–30 min (Rothman et al., 2001; Baumann et al., 2012). Longer periods of uptake inhibition might be expected to shift dose-response curves to the left, increasing apparent drug potency.

In conclusion, this study reveals distinct differences in IC_50_ values when different uptake inhibition assays or cell lines are used. These differences are, at least partially, the result of differences in individual kinetic parameters of the examined transporters. Strikingly, reliable comparison between cell systems and synaptosomes was not possible due to differences in IC_50_ values that are in the range of one order of magnitude more potent in synaptosomes. The binding data were more consistent between different cell lines, yet varied slightly with variations in kinetic parameters. These differences affect the interpretation of *in vitro* results, especially for substances with relevant interaction with all MATs, such as cocaine, MDMA, or mephedrone. Given the limitations with comparing data across laboratories and assays systems, we conclude that suitable reference compounds must be included when investigating the transporter pharmacology of new compounds of interest.

Given the differences in potency measures across methods and various cell lines demonstrated herein, it is essential to include established reference compounds when investigating the in vitro effects of new compounds of interest. MDMA would be a suitable reference compound, as it exerts entactogenic effects in human users and is not associated with high abuse liability (Nichols, 1986), indicating a distinct serotonergic vs. dopaminergic profile in addition to potent NET inhibition (Ritz et al., 1987; Kuhar et al., 1991; Wee et al., 2005; Wee and Woolverton, 2006). Amphetamine could be used as a substance with a distinct dopaminergic vs. serotonergic activity (Simmler et al., 2013; Luethi et al., 2019b). The effect of both substances has been described and compared in controlled clinical studies in humans (Holze et al., 2020). Considering that results from various laboratories cannot be easily compared, it is essential to provide precise methodological details and include reference compounds with established pharmacology to give appropriate context for data interpretation.

## Data Availability Statement

All datasets presented in this study are included in the article/**Supplementary Material**.

## Author Contributions

MI, ML, GL, MB, HS, and DL designed the study. MI, JM, MH, KJ, and DL conducted the experiments. MI, MB, HS, and DL analyzed data. MI and DL wrote the manuscript with significant input from all other authors.

## Funding

This work was supported by grants of the Swiss Federal Office of Public Health (BAG; grant No. 16.921318 to ML), the National Institute on Drug Abuse, National Institutes of Health (grant No. DA 00523 to MB), the Austrian Science Fund (FWF; grant No. F35-B06 to HS), the Vienna Science and Technology Fund (WWTF; grant No. CS15-033 to HS), and the Swiss National Science Foundation (SNF; grant No. P2BSP3_181809 to DL).

## Conflict of Interest

The authors declare that the research was conducted in the absence of any commercial or financial relationships that could be construed as a potential conflict of interest.

The reviewer SB declared a past co-authorship with several of the authors ML, MB, HS, and DL to the handling editor.

## Abbreviations

MAT, monoamine transporter; SERT, serotonin transporter; 5-HT, 5-hydroxytryptamine (serotonin); DAT, dopamine transporter; NET, norepinephrine transporter; HEK 293 cell, human embryonic kidney 293 cell; MDPV, 3,4-methylenedioxypyrovalerone; MDMA, 3,4-methylenedioxymethamphetamine; DMEM, Dulbecco's modified Eagle's medium; PBS, phosphate-buffered saline; PDL, poly-D-lysine; KHB, Krebs HEPES buffer; KRB, Krebs-Ringer Bicarbonate buffer; MPP^+^, 1-methyl-4-phenylpyridinium; CFT, 2-β-carbomethoxy-3-β-(4-fluorophenyl)tropane (WIN 35,428); IC50, half-maximum inhibitory concentration; K_m_, Michaelis–Menten constant; V_max_, maximum rate; K_d_, equilibrium binding constant; Bmax, maximum specific binding.
