# Corrigendum: Cell-Based Radiotracer Binding and Uptake Inhibition Assays: A Comparison of *In Vitro* Methods to Assess the Potency of Drugs That Target Monoamine Transporters

**DOI:** 10.3389/fphar.2022.878641

**Published:** 2022-03-16

**Authors:** Marija Ilic, Julian Maier, Marion Holy, Kathrin Jaentsch, Matthias E. Liechti, Gert Lubec, Michael H. Baumann, Harald H. Sitte, Dino Luethi

**Affiliations:** ^1^ Institute of Pharmacology, Center for Physiology and Pharmacology, Medical University of Vienna, Vienna, Austria; ^2^ Department of Pharmaceutical Chemistry, Faculty of Life Sciences, University of Vienna, Vienna, Austria; ^3^ Neuroproteomics, Paracelsus Private Medical University, Salzburg, Austria; ^4^ Division of Clinical Pharmacology and Toxicology, Department of Biomedicine, University Hospital Basel and University of Basel, Basel, Switzerland; ^5^ Designer Drug Research Unit, Intramural Research Program, National Institute on Drug Abuse, National Institutes of Health, Baltimore, MD, United States

**Keywords:** monoamine, transporter, radiotracer, stimulant, synaptosomes, HEK 293

Julian Maier was not included as an author in the published article. The corrected **Author Contributions** appears below.

“MI, ML, GL, MB, HS, and DL designed the study. MI, JM, MH, KJ, and DL conducted the experiments. MI, MB, HS, and DL analyzed data. MI and DL wrote the manuscript with significant input from all other authors.”

The authors apologize for this error and state that this does not change the scientific conclusions of the article in any way. The original article has been updated.

