# Supplementary material for: Cell-Based Radiotracer Binding and Uptake Inhibition Assays: A Comparison of In Vitro Methods to Assess the Potency of Drugs That Target Monoamine Transporters
Source: Front Pharmacol. 2020 May 19;11:673. doi: 10.3389/fphar.2020.00673 (PMC7248194; doi:10.3389/fphar.2020.00673)
Supplement: Supplementary file 1 [file DataSheet_1.pdf]

Cell-based radiotracer binding and uptake inhibition assays: a comparison of in vitro methods to assess the potency of drugs that target monoamine transporters

Frontiers in Pharmacology

Marija Ilic, Marion Holy, Kathrin Jaentsch, Matthias E. Liechti, Gert Lubec, Michael H. Baumann, Harald H. Sitte, and Dino Luethi

Supplemental data

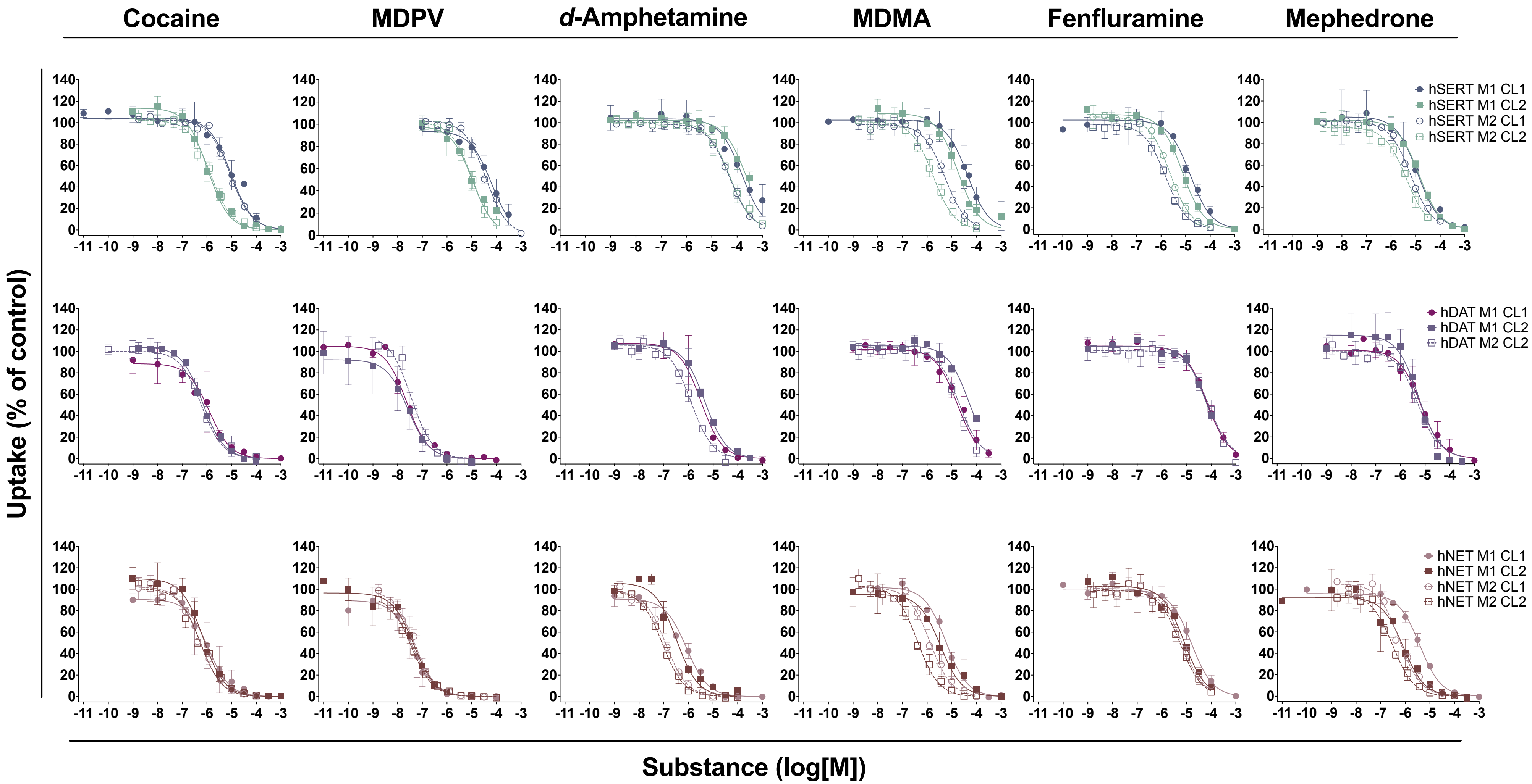

Cocaine
MDPV
d-Amphetamine
MDMA
Fenfluramine
Mephedrone
Uptake (% of control)
Substance (log[M])
hSERT M1 CL1
hSERT M1 CL2
hSERT M2 CL1
hSERT M2 CL2
hDAT M1 CL1
hDAT M1 CL2
hDAT M2 CL2
hNET M1 CL1
hNET M1 CL2
hNET M2 CL1
hNET M2 CL2

Supplementary Figure 1. Uptake inhibition at HEK 293 cells expressing human SERT, DAT, and NET. Concentration-response curves for the inhibition of specific [^{3}H]-5-HT (upper section), [^{3}H]-dopamine (middle section) and [^{3}H]-MPP$^+$ or [^{3}H]-norepinephrine (lower section) uptake by six stimulant substances. Data shown are the mean ± SD of at least three independent experiments performed in triplicates expressed as a % of control uptake. CL1 – cell line 1, CL2 – cell line 2, M1 – method 1 (adherent cells), M2 – method 2 (cells in suspension).

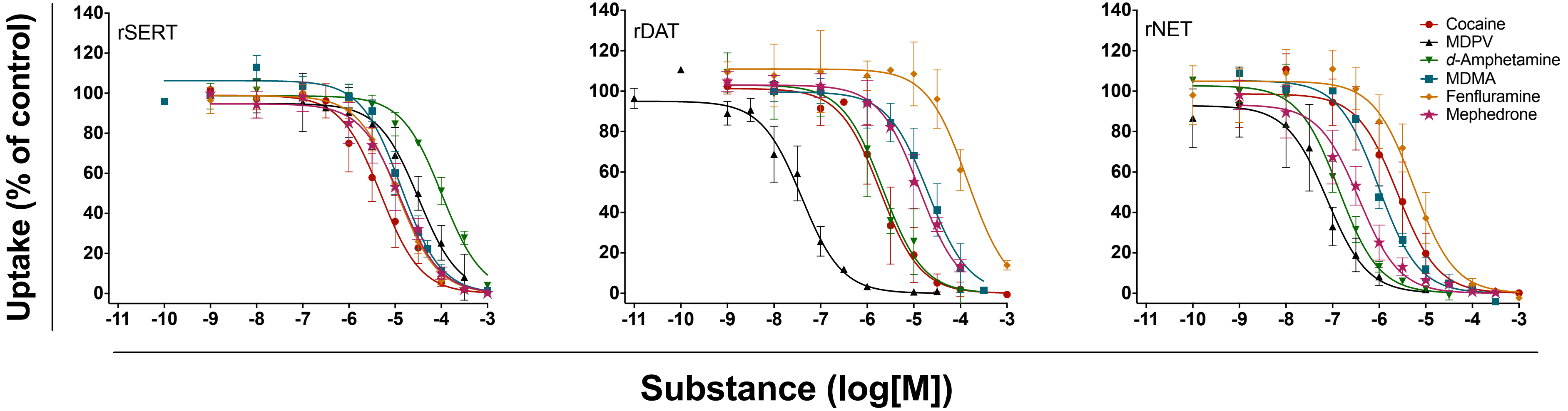

Supplementary Figure 2. Uptake inhibition at HEK 293 cells expressing rat SERT, DAT, and NET. Concentration-response curves for the inhibition of specific [^{3}H]-5-HT (left), [^{3}H]-dopamine (middle) and [^{3}H]-MPP$^+$ (right) uptake by six stimulant substances. Data shown are the mean ± SD of at least three independent experiments performed in triplicates expressed as a % of control uptake.

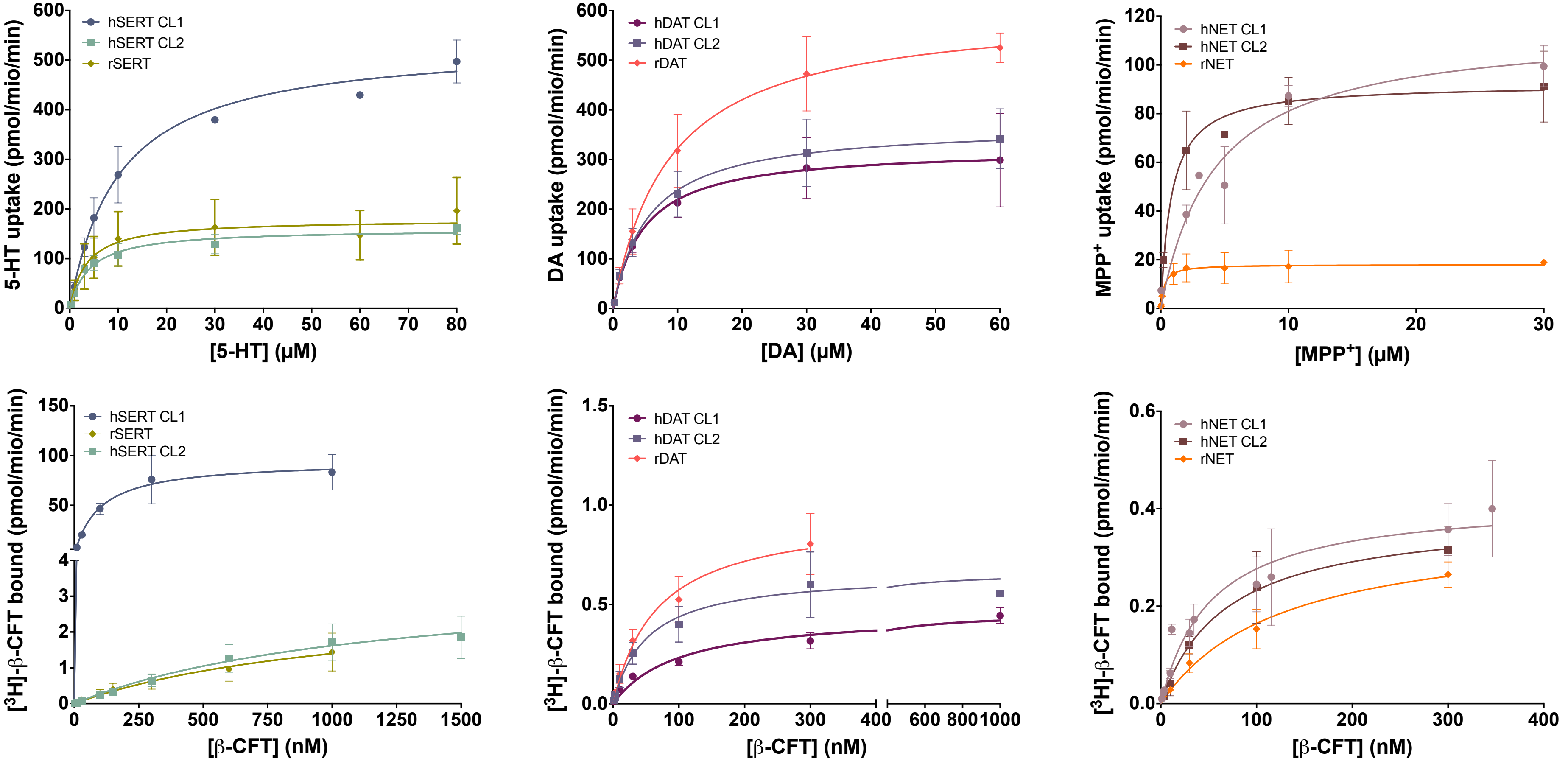

Supplementary Figure 3. Substrate saturation and radioligand binding saturation. Saturation plots of [^{3}H]-5-HT, [^{3}H]-dopamine and [^{3}H]-MPP$^+$ uptake in HEK 293 cells expressing human or rat SERT, DAT or NET, respectively (upper section). Whole-cell β-CFT binding to HEK 293 cells

expressing human or rat SERT, DAT or NET (lower section). Data shown are the mean ± SD of at least three independent experiments performed in triplicates.

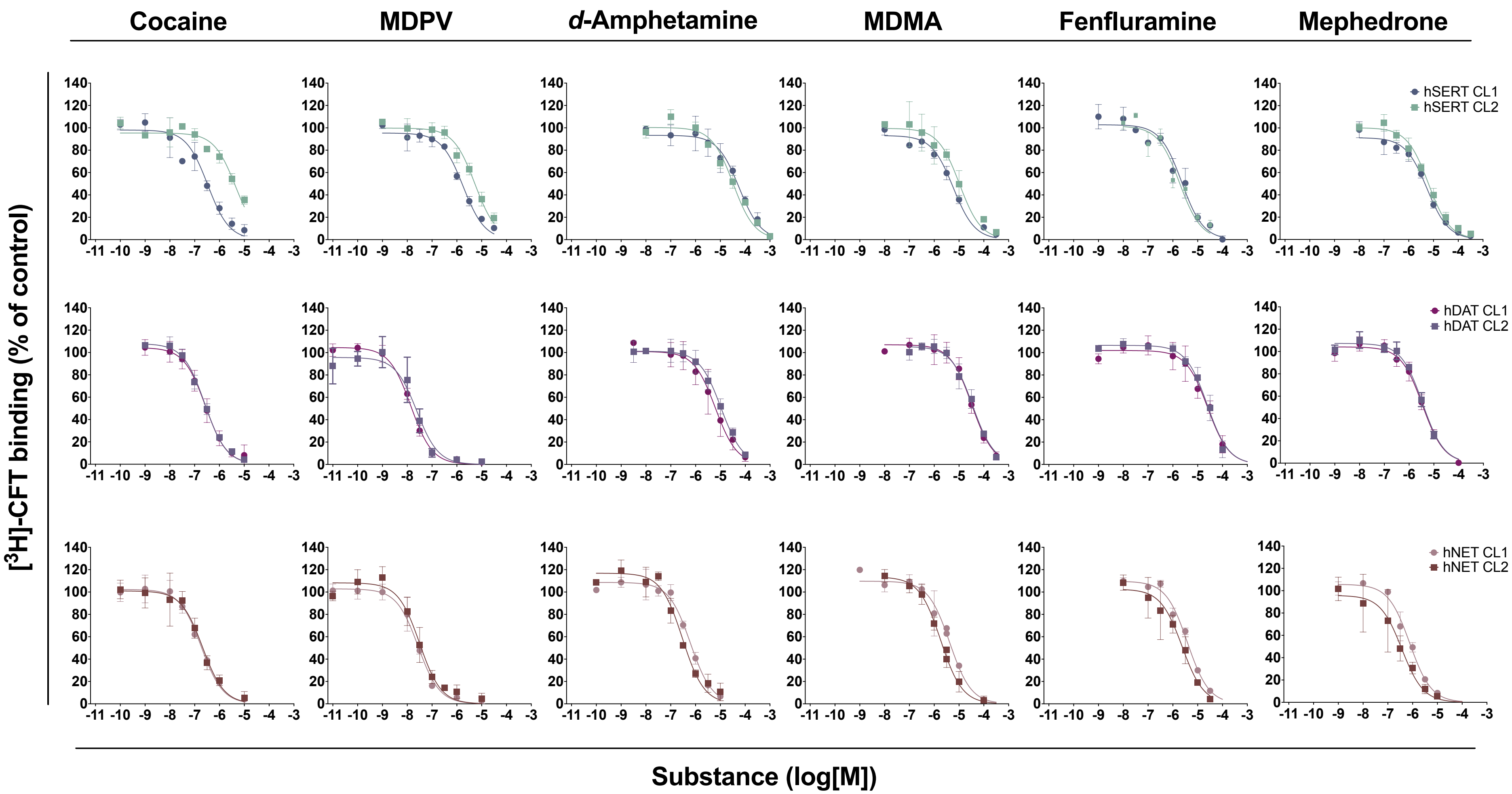

Cocaine
MDPV
d-Amphetamine
MDMA
Fenfluramine
Mephedrone
hSERT CL1
hSERT CL2
hDAT CL1
hDAT CL2
hNET CL1
hNET CL2
[3H]-CFT binding (% of control)
Substance (log[M])

Supplementary Figure 4. Radioligand binding inhibition at HEK 293 cells expressing human SERT, DAT or NET. Concentration-response curves for the inhibition of [^{3}H]-CFT binding to whole-cells. Data shown are the mean ± SD of at least three independent experiments performed in triplicates.

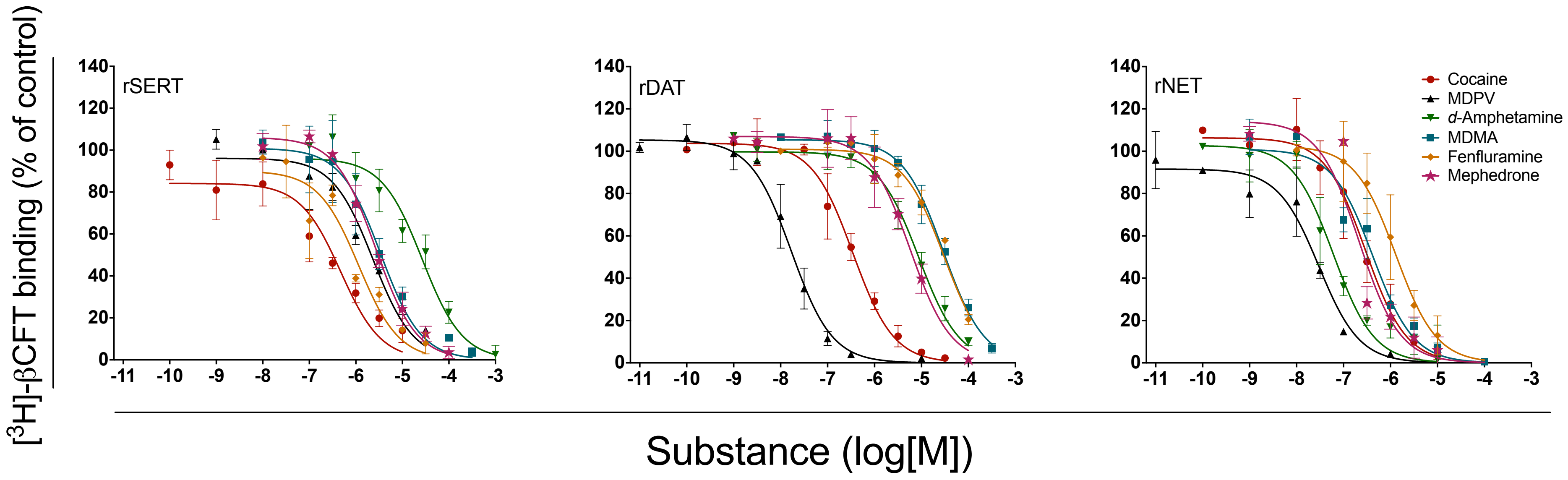

Supplementary Figure 5. Radioligand binding inhibition at HEK 293 cells expressing rat SERT, DAT or NET. Concentration-response curves for the inhibition of [^{3}H]-CFT binding to whole-cells. Data shown are the mean ± SD of at least three independent experiments performed in triplicates.

Supplementary Table 1. Comparison of two uptake inhibition methods in HEK 293 cell lines stably expressing human MATs. Unpaired two-tailed t-test with Welch´s correction for comparisons of unequal variances. Color gradient corresponds to the level of significance; white – not significant to dark gray – significant.

	Cocaine	MDPV	*d*-Amphetamine	MDMA	Fenfluramine	Mephedrone
			P value			
**Cell line 1**			SERT			
Method 1<br>Method 2	0.9114	0.2484	0.0138	0.0024	0.0025	0.2361
			NET			
Method 1<br>Method 2	0.4781	>0.9999	<0.0001	0.0176	0.0756	0.0236
**Cell line 2**			SERT			
Method 1<br>Method 2	0.5341	>0.9999	0.0029	0.0041	0.0087	0.0032
			DAT			
Method 1<br>Method 2	0.1649	0.2199	<0.0001	0.0003	0.0834	0.4820
			NET			
Method 1<br>Method 2	0.1784	0.1963	0.0234	0.0461	0.2681	0.1286

Supplementary Table 2. Comparison of inhibition profiles at two HEK 293 cell lines stably expressing human MATs. Unpaired two-tailed t-test with Welch´s correction for comparisons of unequal variances. Color gradient corresponds to the level of significance; white – not significant to dark gray – significant.

	**Cocaine**	**MDPV**	***d*-Amphetamine**	**MDMA**	**Fenfluramine**	**Mephedrone**
			**P value**			
**Method 1**			**SERT**			
Cell line 1<br>Cell line 2	0.0002	0.0193	0.0996	0.0145	0.0032	0.4478
			**DAT**			
Cell line 1<br>Cell line 2	0.1226	0.0887	0.1889	0.0057	0.4337	0.7558
			**NET**			
Cell line 1<br>Cell line 2	0.6570	>0.9999	0.0009	0.0493	0.1183	0.0008
**Method 2**			**SERT**			
Cell line 1<br>Cell line 2	0.02	<0.0001	0.8299	0.0199	0.0002	0.0354
			**NET**			
Cell line 1<br>Cell line 2	>0.9999	0.1963	0.6560	0.0004	0.6175	0.0003

Supplementary Table 3. Inhibition profile comparison of three HEK 293 cell lines stably expressing human or rat MATs, assayed by Method 1. One-way ANOVA followed by Tukey´s post-test. Color gradient corresponds to the level of significance; white – not significant to dark gray – significant.

	**Cocaine**	**MDPV**	***d*-Amphetamine**	**MDMA**	**Fenfluramine**	**Mephedrone**
				**P value**		
**Method 1**				**SERT**		
Cell line 1<br>Cell line 2<br>Cell line 3	0.0002	0.0248	0.023	0.0022	0.0078	0.6921
				**DAT**		
Cell line 1<br>Cell line 2<br>Cell line 3	0.0708	0.4042	0.0348	0.0076	<0.0001	0.0051
				**NET**		
Cell line 1<br>Cell line 2<br>Cell line 3	<0.0001	0.003	0.0004	<0.0001	0.0021	<0.0001

Supplementary Table 4. Comparison of binding inhibition profiles at two HEK 293 cell lines stably expressing human MATs. Unpaired two-tailed t-test with Welch´s correction for comparisons of unequal variances. Color gradient corresponds to the level of significance; white – not significant to dark gray – significant.

	**Cocaine**	**MDPV**	***d*-Amphetamine**	**MDMA**	**Fenfluramine**	**Mephedrone**
				**P value**		
				**SERT**		
Cell line 1<br>Cell line 2	0.0187	0.0013	0.0065	0.0392	0.2026	0.9170
				**DAT**		
Cell line 1<br>Cell line 2	0.8400	0.0531	0.0564	0.7563	0.8804	>0.9999
				**NET**		
Cell line 1<br>Cell line 2	0.4178	0.6108	0.0034	0.0059	0.3149	0.0034

Supplementary Table 5. Binding inhibition profile comparison of three HEK 293 cell lines stably expressing human or rat MATs. One-way ANOVA followed by Tukey´s post-test. Color gradient corresponds to the level of significance; white – not significant to dark gray – significant.

	**Cocaine**	**MDPV**	***d*-Amphetamine**	**MDMA**	**Fenfluramine**	**Mephedrone**
			**P value**			
**Method 1**			**SERT**			
Cell line 1<br>Cell line 2<br>Cell line 3	<0.0001	0.001	0.0002	0.0008	0.0182	0.0299
			**DAT**			
Cell line 1<br>Cell line 2<br>Cell line 3	0.3111	0.1343	0.1087	0.5627	0.3830	0.0032
			**NET**			
Cell line 1<br>Cell line 2<br>Cell line 3	0.2935	0.8553	<0.0001	<0.0001	0.012	0.0004

Supplementary Table 6. Kinetic parameters for the uptake and binding of radioligands in adherent transporter-transfected HEK 293 cells. Unpaired two-tailed t-test with Welch´s correction for comparisons of unequal variances. Color gradient corresponds to the level of significance; white – not significant to dark gray – significant.

	K_m	V_{max}	K_d	B_{max}
	**P value**			
	**Cell line 1/Cell line 2**			
hSERT	0.0817	0.0002	0.0491	0.0261
hDAT	0.7175	0.4513	0.3852	0.1546
hNET	0.0163	0.0008	0.3300	>0.9999

Supplementary Table 7. Kinetic parameters for the uptake and binding of radioligands in human and rat adherent transporter-transfected HEK 293 cells. One-way ANOVA followed by Tukey´s post-test. Color gradient corresponds to the level of significance; white – not significant to dark gray – significant.

	K_m	V_{max}	K_d	B_{max}
	P value			
	Cell line 1/Cell line 2/Cell line 3			
SERT	0.0126	<0.0001	0.0012	0.0001
DAT	0.1786	0.0021	0.2244	0.0146
NET	<0.0001	<0.0001	0.0972	0.4149
